# Contrasting effects of whole-body and hepatocyte-specific deletion of the RNA polymerase III repressor *Maf1* in the mouse

**DOI:** 10.3389/fmolb.2023.1297800

**Published:** 2023-12-05

**Authors:** Gilles Willemin, François Mange, Viviane Praz, Séverine Lorrain, Pascal Cousin, Catherine Roger, Ian M. Willis, Nouria Hernandez

**Affiliations:** ^1^ Center for Integrative Genomics, Faculty of Biology and Medicine, University of Lausanne, Lausanne, Switzerland; ^2^ Lausanne Genomic Technologies Facility, Faculty of Biology and Medicine, University of Lausanne, Lausanne, Switzerland; ^3^ Protein Analysis Facility, Faculty of Biology and Medicine, University of Lausanne, Lausanne, Switzerland; ^4^ Department of Biochemistry, Albert Einstein College of Medicine, Bronx, NY, United States; ^5^ Department of Systems and Computational Biology, Albert Einstein College of Medicine, Bronx, NY, United States

**Keywords:** transcription repressor, metabolic regulation, lipogenesis, angiogenin, growth hormone, ChIPseq, RNAseq, proteomics

## Abstract

MAF1 is a nutrient-sensitive, TORC1-regulated repressor of RNA polymerase III (Pol III). MAF1 downregulation leads to increased lipogenesis in *Drosophila melanogaster*, *Caenorhabditis elegans*, and mice. However, *Maf1* ^*−/−*^ mice are lean as increased lipogenesis is counterbalanced by futile pre-tRNA synthesis and degradation, resulting in increased energy expenditure. We compared Chow-fed *Maf1* ^*−/−*^ mice with Chow- or High Fat (HF)-fed *Maf1* ^*hep−/−*^ mice that lack MAF1 specifically in hepatocytes. Unlike *Maf1* ^*−/−*^ mice, *Maf1* ^*hep−/−*^ mice become heavier and fattier than control mice with old age and much earlier under a HF diet. Liver ChIPseq, RNAseq and proteomics analyses indicate increased Pol III occupancy at Pol III genes, very few differences in mRNA accumulation, and protein accumulation changes consistent with increased lipogenesis. Futile pre-tRNA synthesis and degradation in the liver, as likely occurs in *Maf1* ^*hep−/−*^ mice, thus seems insufficient to counteract increased lipogenesis. Indeed, RNAseq and metabolite profiling indicate that liver phenotypes of *Maf1* ^*−/−*^ mice are strongly influenced by systemic inter-organ communication. Among common changes in the three phenotypically distinct cohorts, Angiogenin downregulation is likely linked to increased Pol III occupancy of tRNA genes in the Angiogenin promoter.

## 1 Introduction

MAF1 is a physiologically important repressor of Pol III transcription conserved in eukaryotes from yeast to mammals (Pluta et al., 2001; Upadhya et al., 2002). MAF1 is repressed by TOR signaling (Huber et al., 2009; Lee et al., 2009; Wei et al., 2009; Wei and Zheng, 2009; Michels et al., 2010) and acts by binding to Pol III on and off its target genes, resulting in decreased Pol III occupancy and transcription (Desai et al., 2005; Reina et al., 2006; Vannini et al., 2010; Orioli et al., 2016; Bonhoure et al., 2020). In yeast, MAF1 is activated to repress Pol III transcription in response to diverse stressors such as nutrient limitation, DNA damage, or secretory pathway defects, and its deletion greatly attenuates or blocks transcriptional repression. It is, however, largely derepressed in rich media (Upadhya et al., 2002). In mammalian cells, MAF1 represses Pol III transcription not only under stress conditions such as rapamycin treatment but also under optimal growth conditions, albeit to a lesser extent (Reina et al., 2006; Orioli et al., 2016). Indeed, the livers of *Maf1* ^*−/−*^ mice display higher Pol III occupancy of Pol III genes in both fasted and refed states and the levels of short-lived precursor tRNAs, which reflect transcription activity, are elevated in multiple tissues (Bonhoure et al., 2020). Mammalian MAF1 thus keeps Pol III transcription in check under many different conditions.

Decreased MAF1 levels have been linked to metabolic disturbances in several systems. In *Drosophila melanogaster* and *Caenorhabditis elegans*, MAF1 reduction by RNAi resulted in increased tRNA production, translation, body size, and fat accumulation (Rideout et al., 2012; Khanna et al., 2014; Hammerquist et al., 2021). Similarly, lowered MAF1 levels were associated with increased lipid accumulation in mouse hepatoma cells (Palian et al., 2014), and increased insulin-like signaling in *D. melanogaster* (Rideout et al., 2012). In the fly, the effects could be mimicked by overproduction of the initiator tRNA^Met^ or by *Maf1* knockdown in just the fat body, the main larval endocrine organ, suggesting that increased translation of certain mRNAs in the fat body signaled increased expression of brain-derived insulin-like peptides and thus increased insulin signaling (Rideout et al., 2012). In the worm, MAF1 reduction affected expression of vitellogenin genes as well as Pol II genes involved in lipid biosynthesis through an undefined mechanism (Khanna et al., 2014), and in a mouse hepatocyte cell line, MAF1 was shown to negatively regulate Fasn and Acc expression, in the first case through a 178 base pair promoter region (Palian et al., 2014), consistent with a previous observation that MAF1 binds to and represses certain Pol II promoters (Johnson et al., 2007).

In contrast, *Maf1* whole-body knockout (*Maf1* ^*−/−*^) mice under a Chow or a High Fat (HF) diet were lighter, with substantially less fat than control mice even though they display higher lipogenesis (Bonhoure et al., 2015). This results from various metabolic inefficiencies, particularly increased transcription of tRNA genes resulting in increased levels of precursor, but not mature, tRNAs. This, together with metabolomics analyses pointing to elevated nucleotide synthesis and RNA turnover (Willis et al., 2018), pinpointed futile pre-tRNA synthesis and degradation as an important driver of the wasteful use of metabolic energy. *Maf1* ^*−/−*^ mice also displayed a slight reduction of overall translation in the liver (Bonhoure et al., 2020). Thus, in several systems, MAF1 is linked to lipid metabolism through effects on Pol III transcription, Pol II transcription, or both.

The liver is a metabolic organ with vital roles in handling and (re)distribution of metabolites involved in the generation of cellular energy. To explore further the role of MAF1 in mouse metabolism, we have analyzed Chow- or HF-fed mice lacking *Maf1* specifically in hepatocytes (*Maf1* ^*hep−/−*^ mice) and compared them with Chow-fed *Maf1* ^*−/−*^ mice. Unlike *Maf1* ^*−/−*^ mice, *Maf1* ^*hep−/−*^ mice are heavier and fattier than control (Ctrl) mice and their livers show protein accumulation changes consistent with increased lipogenesis. Common changes occurring in all three cohorts are suggestive of an alteration in Growth Hormone (GH) levels or release pattern, and a decrease in Angiogenin that likely results directly from increased Pol III transcription. The data also reveal substantial systemic effects on liver mRNA and metabolism in *Maf1* ^*−/−*^ mice, reflecting the importance of MAF1 function in whole-body metabolic economy and underscoring how a molecular change can have very different outcomes when present in a single cell type (hepatocytes) or within all cell types of an entire organism.

## 2 Materials and methods

### 2.1 Animals

**FIGURE 1:**
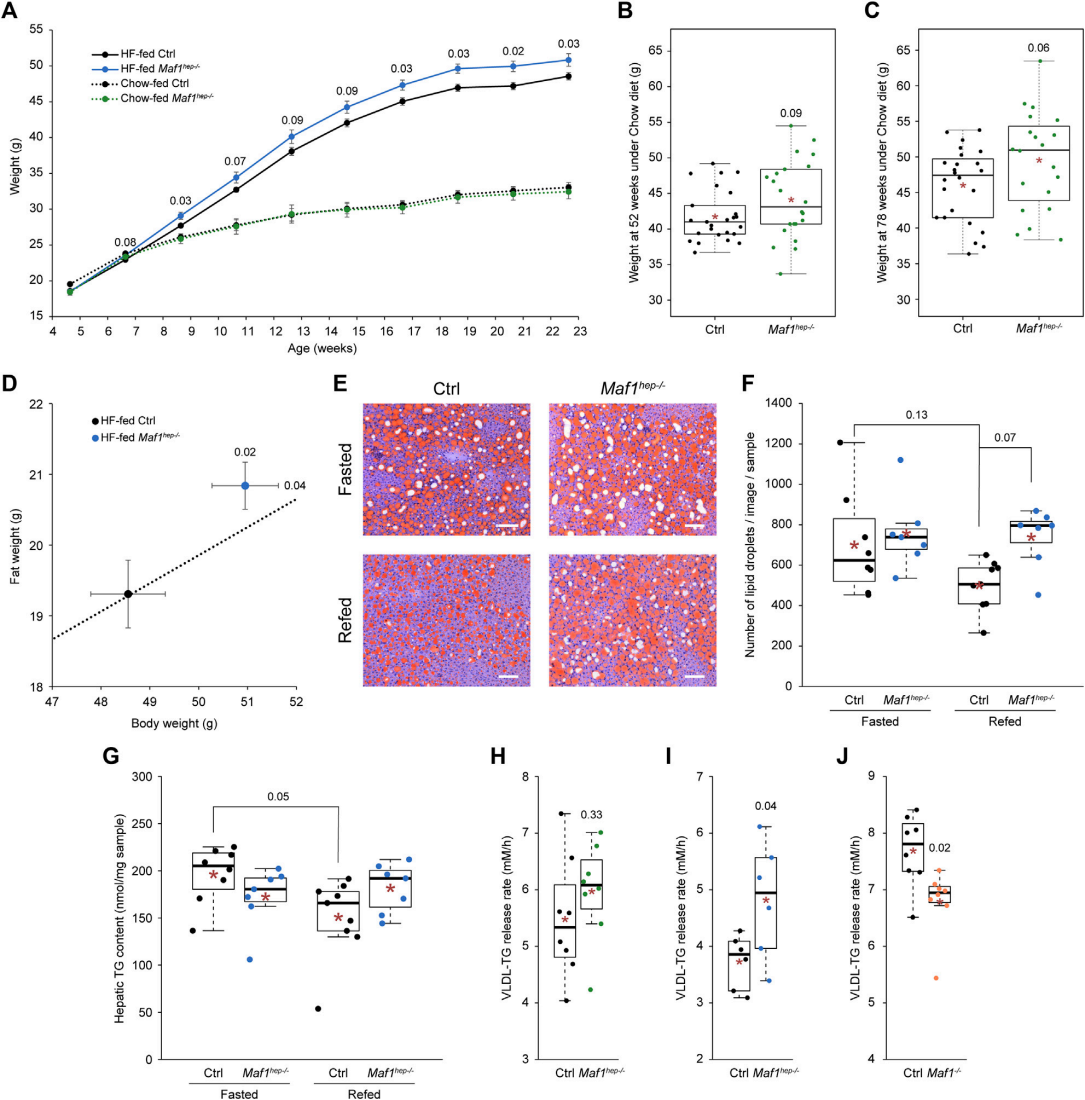
Weight and lipid metabolism in *Maf1* ^*hep−/−*^ mice. **(A)** Weight of Ctrl and *Maf1* ^*hep−/−*^ mice fed either a Chow (dotted black and green lines, respectively) or a HF (solid black and blue lines, respectively) diet. Chow-fed Ctrl *n* = 19 and *Maf1* ^*hep−/−*^ *n* = 9; HF-fed Ctrl *n* = 17 and *Maf1* ^*hep−/−*^ *n* = 14. Means +/- SEM are indicated. **(B, C)** Weight of Chow-fed Ctrl and *Maf1* ^*hep−/−*^ mice aged 52 weeks **(B)**, Ctrl *n* = 25 and *Maf1* ^*hep−/−*^ *n* = 22, and 78 weeks **(C)**, Ctrl *n* = 24 and *Maf1* ^*hep−/−*^ *n* = 20. **(D)** Whole body adiposity at 24 weeks of age in HF-fed Ctrl and *Maf1* ^*hep−/−*^ mice. Dotted line: extrapolation of the mean ratio of Ctrl mice fat weight/body weight; the ratio in HF-fed *Maf1* ^*hep−/−*^ is higher than in Ctrl mice (*p*-value = 0.11). Ctrl *n* = 17 and *Maf1* ^*hep−/−*^ *n* = 14. **(E)** Neutral lipid Oil Red O staining of liver slices from fasted or refed 24 week-old HF-fed Ctrl and *Maf1* ^*hep−/−*^ mice. White bars: 100 µm. **(F)** Lipid droplet quantification from four representative images as in **(E)** for each of seven to nine samples per genotype. Fasted Ctrl *n* = 8 and *Maf1* ^*hep−/−*^ *n* = 7; Refed Ctrl *n* = 9 and *Maf1* ^*hep−/−*^ *n* = 7. **(G)** Hepatic TG content in fasted or refed 24 week-old HF-fed Ctrl and *Maf1* ^*hep−/−*^ mice. *n* values are identical as in **(F)**. **(H)** VLDL-TG release rate in 14–15 week-old Chow-fed Ctrl and *Maf1* ^*hep−/−*^ mice. *n* = 8 for each group. **(I)** VLDL-TG release rate in 14–15 week-old HF-fed Ctrl and *Maf1* ^*hep−/−*^ mice. *n* = 6 for each group. **(J)** VLDL-TG release rate in 14 week-old Chow-fed Ctrl and *Maf1* ^*−/−*^ mice. *n* = 8 for each group. In **(B**, **C)** and **(F**–**J)**, the boxes encompass upper and lower quartile, the whiskers extend to 1.5 times the interquartile range of the box. Red star, mean; horizontal bar, median. Numbers inside figures correspond to *p*-values.

We generated *Maf1* ^*hep−/−*^ mice by crossing C57BL/6J homozygous mice with loxP sites in intron 1 and exon 8 of the *Maf1* gene (Bonhoure et al., 2015) with C57BL/6J mice heterozygous for an *Alb-Cre* transgene expressing *Cre* recombinase from the *Albumin* promoter (Jax strain #:003574) (Postic et al., 1999). F1 mice were crossed, after which homozygous floxed females were crossed with homozygous floxed males hemizygous for the *Alb-Cre* transgene. Thus, mothers did not carry the *Maf1* deletion, eliminating the possibility of any effect on fetal development and pup nurturing. F2 mice were viable and fertile, showing a Mendelian ratio for sex and *Alb-Cre* transgene transmission (Supplementary Table S10). Experimental animals were homozygous for the floxed *Maf1* allele and either lacked (Ctrl mice), or were hemizygous for (*Maf1* *^hep−/−^* mice), the *Alb-Cre* transgene. In some of the experiments in Figure 1, Ctrl mice were heterozygous for the floxed *Maf1* allele. *Maf1* ^*−/−*^ mice and PCR genotyping have been described previously (Bonhoure et al., 2015).

All protocols were approved by the Veterinary Office of the Canton of Vaud. Male mice were fed either a Chow diet (Kliba Nafag diet #3436) or a High Fat diet (60% of calories coming from lard–BioServ diet #F3282) from 4 to 5 weeks of age. Animals were housed under 12 h light/dark conditions, at a constant temperature of 22°C and fed *ad-libitum* with free access to water. Animals were weighed at the same time of day for long-term experiments. Body composition (measured by EchoMRI) and length from nose to anus were measured on Isoflurane-anesthetized animals.

### 2.2 Serum parameters measurements

Serum parameters were measured on a Roche Cobas C111 automatic analyzer with proprietary Roche Glucose, ALT, AST, LDH, TG and cholesterol kits, and a Fujifilm-Wako NEFA (FFA) kit. Insulinemia was assessed with a Mouse Insulin ELISA kit (Mercodia 10-1247-01). For the HF-fed cohort only, fasting glycemia was assessed with a Roche Breeze2 glucometer directly on blood samples–obtained by tail vein incision.

### 2.3 Oil Red O histology

Liver samples were frozen in OCT cryo-embedding medium for Oil Red O staining. Lipid droplets were counted with the Analyze particles function of ImageJ on four images per mouse.

### 2.4 Hepatic lipids

Hepatic lipids were extracted by a modified Folch method with beads and methyl-*tert*-butyl ether (Abbott et al., 2013) with the following changes: ∼50 mg of liver were homogenized in 462 µL of methanol, incubated for an hour at room temperature, and mixed with 385 µL of ultrapure water. The sample was dried and resuspended in 300 µL of chloroform. A third of each extract was then mixed with 100 µL of chloroform/Triton-X100 5%, dried, and resuspended in 500 µL of ultrapure water. TG, FFA and total cholesterol content was then assessed with a Roche Cobas C111.

### 2.5 VLDL-TG release test

Mice were weighed in the morning and then fasted for 5 h to remove any TG-containing chylomicrons from the bloodstream. A tail blood sample (∼50 µL) was collected before retro-orbital IV injection of Tyloxapol (Triton, WR-1339) at 600 mg/kg mouse (10% in a 0.9% NaCl solution) under Isoflurane anesthesia, to block peripheral clearance of TG-containing lipoproteins. About 50 µL of blood/mouse was then harvested at 40, 80, 120 and 160 min after injection, and TG concentration was measured with a Roche Cobas C111 to determine rates of VLDL-TG release.

### 2.6 ChIPseq

ChIPseq and Pol III occupancy score calculations were as previously described (Bonhoure et al., 2014; Mange et al., 2017), except for the following changes: Reads were trimmed with TrimGalore v 0.6.4 and aligned to the mouse genome assembly Mm10 with STAR v 2.5.0a. Pol III gene regions were scored extended to 50 nucleotides on both 5′ and 3′ ends. The cutoff for unoccupied Pol III regions was calculated as previously described (Renaud et al., 2014).

Significantly differentially occupied Pol III genes were identified from the scores with lmFit of the Limma package by fitting a linear model on the selected samples. *p*-values calculated using eBayes were adjusted for multiple testing using the Benjamini and Hochberg correction.

### 2.7 RNAseq

RNAseq was as previously described (Bonhoure et al., 2020), except that samples for the HF-fed *Maf1* ^*hep−/−*^ cohort were loaded on two lanes of a HiSeq 4000 flow cell and sequenced at 150 cycles. Reads were trimmed and aligned as above. Transcripts were quantified with rsem v 1.3.068. The resulting count matrix was used for subsequent analysis in R, on the three cohorts independently; gene filtering was based on the rule of 1 count per M (cpm) in at least 1 sample, library size was scaled with TMM normalization (EdgeR package v 3.32.1) (Robinson et al., 2010) and log-transformation was done with the Limma voom function (Ritchie et al., 2015). Unwanted variation was removed with RUVr (RUVseq package v 1.24.0) (Risso et al., 2014). Statistically significant differentially expressed genes were identified from log-transformed, TMM-scaled values as above. *p*-values were calculated as above. RUVr-computed factors were added in the design matrix.

### 2.8 Metabolomics

Twenty-two week old male mice were sacrificed after an overnight fasting and 4 hours refeeding. The liver was rapidly collected and freeze-clamped in liquid nitrogen after removal of the gall bladder. Targeted absolute quantitative analysis of metabolites in central metabolism was performed by capillary electrophoresis-mass spectrometry using the C-SCOPE platform (Human Metabolome Technologies Inc.).

### 2.9 Proteomics

Samples were precipitated with cold acetone and digested with a modified iST method (Kulak et al., 2014). Briefly, benzonase was added in miST lysis buffer (1% Sodium deoxycholate, 100 mM Tris pH8.6, 10 mM DTT) to digest nucleic acids, reduced disulfides were alkylated, and samples were digested with Trypsin/LysC mix. Ethyl acetate 100%/1% TFA was then added to the digests, the aqueous phases were collected and desalted on a strong cation exchange (SCX) plate. Eluates were labelled with TMT-10 plex reagent, the TMT multiplex samples were desalted and fractionated into 24 fractions by off-gel focusing (Geiser et al., 2011). LC-MS/MS analyses of TMT fractions were carried out on a Fusion Tribrid Orbitrap mass spectrometer (Thermo Fisher) interfaced to an Ultimate 3000 RSLCnano HPLC system (Dionex). Peptides were separated on a reversed-phase custom packed C18 column (75 µm ID, 100 Å, Reprosil-Pur 1.9 µm particles, Dr. Maisch).

Data files were analyzed with MaxQuant 1.6.3.4 (Cox and Mann, 2008; Cox et al., 2011), using *Mus musculus* reference proteome database from UniProt (www.uniprot.org, 29.10.2017 version). Peptide and protein identifications were filtered at 1% FDR. MaxQuant tables were processed with Perseus software for normalization, statistical tests and GO annotation enrichment tests (Cox and Mann, 2012; Tyanova et al., 2016). PCA analysis and weight (45.3 g compared to a mean weight of 32.8 g) identified one mouse (HF-hCT6) as an outlier, which was thus excluded from the analysis. RNAseq and proteomics data were merged using gene symbols as key identifiers.

### 2.10 Capillary-based immunoassays

ACC, phosphorylated ACC, and FAS levels were quantified with the capillary-based immunoassay WES™ system (ProteinSimple) using the 66–440 kDa separation module (ProteinSimple SM-W008) according to the manufacturer’s instructions.

### 2.11 Puromycin experiments

Puromycin incorporation was performed as previously described (Bonhoure et al., 2020), on one cohort of Chow-fed 13–14 week-old *Maf1* ^*hep−/−*^ mice and Ctrl. Liver samples were either used for puromycin immunostaining as previously described (Bonhoure et al., 2020), or homogenized and protein content determined by capillary-based immunoassay as above with anti-puromycin antibodies.

### 2.12 Statistics

Normal distribution of each group of sampled data was assessed in R with the Shapiro-Wilk normality test. When two groups were compared, the equality of variance between them was determined with the F test. When data was determined as representing a normal distribution in the two groups with an equal variance between them, a Student’s t-test was performed. When the data had a non-normal distribution with an equal variance, a Mann-Whitney test was performed, except when the n for the smallest group was lower than 6 in which case a Student’s t-test was performed. When the data had either a normal or a non-normal distribution but with an unequal variance, a Welch *t*-test was performed.

When four groups were compared, the equality of variance of all groups was assessed by a Levene test. When data was determined as representing a normal distribution in all groups with an equal variance between them, a Two-Way ANOVA was performed, followed by Tukey-Kramer *post hoc* tests. When the data in one or more groups had a non-normal distribution with an equal variance, four pairwise Mann-Whitney tests were performed followed by a Bonferroni correction, except when the n for the smallest group was lower than 6 in which case Student t-tests were performed, followed by a Bonferroni correction. When the data had normal or non-normal distributions but with unequal variance between the groups, four pairwise Welch t-tests were performed followed by a Bonferroni correction.

None of our tested datasets were paired. *p*-values thresholds were as follow: <0.05 for significance and <0.15 for tendency.

As described above in the relevant Method sections, standard ChIPseq, RNAseq and Proteomics statistics procedures were used. As for the Metabolomics data, 251 out of 292 groups had a n ≥ 3 and could thus be tested for normality with the Shapiro-Wilk test. Out of these 251, only 12 groups (4.8%) could not be considered normally distributed. Out of the 128 comparisons on which equality of variance between groups could be tested, only 10 showed unequal variance (7.8%). We thus opted to use the Student’s t-test already used in the published data with the *Maf1* ^*−/−*^ mice (Willis et al., 2018) so as to be able to compare directly the *Maf1* ^*−/−*^ and *Maf1* ^*hep−/−*^ data.

## 3 Results

### 3.1 Generation of the *Maf1* ^*hep−/−*^ mice

We generated *Maf1* ^*hep−/−*^ mice by crossing mice with loxP sites flanking the protein coding exons of the *Maf1* gene (Bonhoure et al., 2015) with mice containing an *Alb-Cre* transgene (Postic et al., 1999) whose expression starts in the late fetal stage and marks hepatocyte differentiation (Weisend et al., 2009). Following *Alb-Cre* expression and LoxP-mediated recombination, *Maf1* ^*hep−/−*^ mice lack, specifically in hepatocytes, the same ∼1000 base pairs of genomic DNA as *Maf1* ^*−/−*^ mice (Supplementary Figure S1A). As expected, since hepatocytes constitute about 80% of liver cells, *Maf1* RNA was strongly reduced in liver (Supplementary Figure S1B). Healthspan and lifespan, which were increased in male and female *Maf1* ^*−/−*^ mice, respectively (Bonhoure et al., 2015), were unaffected in male *Maf1* ^*hep−/−*^ mice (Supplementary Figure S1C).

### 3.2 *Maf1* ^*hep−/−*^ mice are heavier than Ctrl mice

Chow-fed Ctrl and *Maf1* ^*hep−/−*^ mice exhibited similar body weights up until 24 weeks of age (Figure 1A). However, from 52 to 78 weeks of age, *Maf1* ^*hep−/−*^ mice tended to be heavier than Ctrl mice (Figures 1B, C). HF-fed *Maf1* ^*hep−/−*^ mice gained more weight than Ctrl mice from 7 weeks of age to become on average 2.5 g heavier by 23 weeks of age (Figure 1A). Fat weight was significantly increased at 24 weeks of age (Figure 1D), and fat percentage relative to total body weight tended to be higher (Figure 1D, Supplementary Figure S1D). In addition, these mice were slightly longer (Supplementary Table S1). Thus, unlike *Maf1* ^−/−^ mice, which were lighter and leaner than Ctrl, *Maf1* ^*hep−/−*^ mice were heavier and fattier especially when on a HF diet.

### 3.3 *Maf1* ^*hep−/−*^ mice display hepatic steatosis and an increased rate of VLDL-TG release

We assessed selected metabolic parameters (Alanine Transaminase (ALT), Aspartate Transaminase (AST), Lactate Dehydrogenase (LDH), triglycerides (TG), Free Fatty Acids (FFA) and total cholesterol) of 24-week old Chow- and HF-fed Ctrl and *Maf1* ^*hep*−/−^ mice after overnight fasting followed or not by 4 h refeeding (Supplementary Table S1). In Chow-fed mice, measured serum parameters were largely similar to Ctrl. The same was true in HF-fed mice except for i) a tendency to lower insulinemia in the fasted state when compared to Ctrl, and ii) no ALT, AST, and LDH decrease after refeeding.

To investigate further differences in lipid metabolism, we measured hepatic TG and FFA, and stained liver slices with Oil Red O to visualize lipid droplets. Chow-fed Ctrl and *Maf1* ^*hep−/−*^ mice displayed similar levels of lipid droplets, TG, and FFA; lipid droplet and TG content were in both cases higher in the fasted state (Supplementary Figures S1E, F), reflecting increased TG synthesis and increased production of very low density lipoprotein (VLDL) for release into the bloodstream and delivery to other organs (Saponaro et al., 2015). Similarly, FFA levels were higher in the fasted state (Supplementary Figure S1G), reflecting higher intake from the bloodstream. Thus, under Chow conditions, the regulation of neutral lipids appeared normal.

HF-fed Ctrl mice had again more lipid droplets (Figures 1E, F) and a higher hepatic TG content (Figure 1G) in fasted than in refed conditions, but in *Maf1* ^*hep−/−*^ mice, both values were high and not significantly different in fasted and refed conditions (Figures 1E–G). Although in the refed state of HF-fed *Maf1* ^*hep−/−*^ mice the liver TG content was not significantly higher than in Ctrl mice (Figure 1G), there was a tendency to increased lipid droplets (Figure 1F). This pattern mirrors that described above for ALT, AST, and LDH serum levels (Supplementary Table S1), consistent with the known correlation between the levels of circulating ALT and AST and hepatic steatosis (Deng et al., 2005; Ganbold et al., 2019). Hepatic FFA content was similar in Ctrl and *Maf1* ^*hep−/−*^ mice and decreased after refeeding, as expected (Supplementary Figure S1H), and hepatic cholesterol levels were similar in all cases (Supplementary Figure S1I).

The putative difference in liver fat content under refed conditions in Ctrl and *Maf1* ^*hep−/−*^ mice suggested an alteration of liver TG release in *Maf1* ^*hep−/−*^ mice. We performed VLDL-TG release rate tests on Chow- and HF-fed Ctrl and *Maf1* ^*hep−/−*^ mice subjected to a mild 5-h fast during the day to deplete TG from sources (mostly chylomicrons) other than VLDL. VLDL-TG release was similar in 14–15 week-old *Maf1* ^*hep−/−*^ and Ctrl mice fed a Chow diet, but was significantly increased in *Maf1* ^*hep−/−*^ mice as compared to Ctrl mice fed a HF diet (Figures 1H, I). This was in contrast to 14 week-old Chow-fed *Maf1* ^*−/−*^ mice, which displayed a decreased VLDL release rate (Figure 1J). Thus, in HF-fed *Maf1* ^*hep−/−*^ mice, there is a tendency to increased liver droplets as compared to Ctrl under refed conditions, and clearly increased VLDL-TG release after mild fasting. This suggests impaired regulation of hepatic lipids in *Maf1* ^*hep−/−*^ mice with an increased flux of lipids through the liver involving increased release into the bloodstream (and delivery to other organs), consistent with the observed higher body weight and adiposity under challenging conditions such as HF diet or old age.

### 3.4 Increased Pol III occupancy in the absence of *Maf1*

**FIGURE 2:**
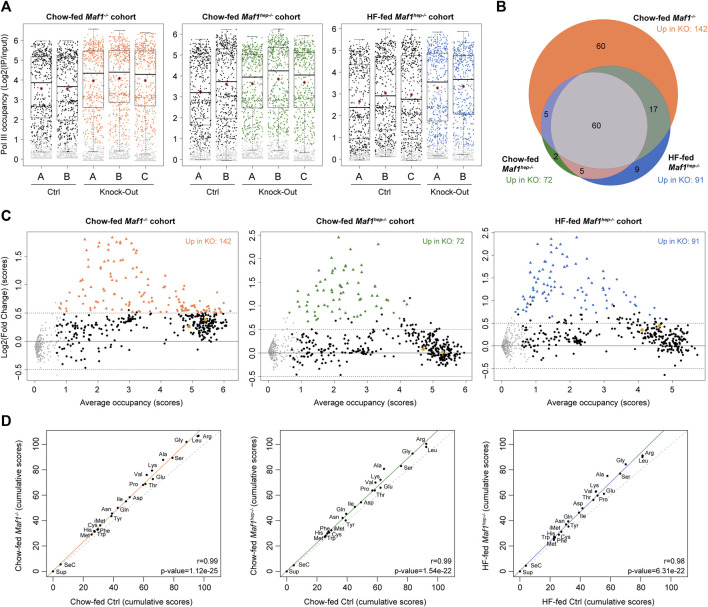
Higher Pol III occupancy in the absence of MAF1. **(A)** Boxplot representation of Pol III loci occupancy score distribution (Log2(IP/input)) for the indicated samples. Grey dots: loci with scores below the cut-off and thus considered unoccupied. The box encompasses upper and lower quartile, the whiskers extend to 1.5 times the interquartile range of the box. Red star, mean; horizontal bar, median. **(B)** Venn diagram (adapted from www.biovenn.nl) showing numbers of loci differentially occupied in the Chow-fed *Maf1* ^*−/−*^, Chow-fed *Maf1* ^*hep−/−*^, HF-fed *Maf1* ^*hep−/−*^ and their respective Ctrl cohorts. All scores were increased. Chow-fed *Maf1* ^*−/−*^: 142 loci, of which 132 tRNAs, 1 tRNA pseudogene, 4 SINEs, 1 non-annotated, Rny1 (Y1 RNA), 2 RnU6 (U6 snRNA), 1 Rn5s (5S RNA). Chow-fed *Maf1* ^*hep−/−*^: 72 loci of which 70 tRNAs, 1 tRNA pseudogene, 1 SINE. HF-fed *Maf1* ^*hep−/−*^: 91 loci of which 88 tRNAs, 1 tRNA pseudogene, 1 SINE, 1 non-annotated. **(C)** MvA plots showing average occupancy scores ((Ctrl scores + *Maf1* knockout score)/2) in the indicated cohorts on the *X*-axes and the log2(FC) on the *Y*-axes. Grey dots as in **(A)**; black dots, loci whose score did not change significantly; colored dots and triangles, loci whose scores changed significantly, with triangles indicating loci that changed in all three cohorts. Oversized gold dots; chr14_293_tRNATyr_GTA+ and chr14_294_tRNAPro_TGG+ tRNA genes. **(D)** Spearman rank correlations of Pol III occupancy cumulated scores for each tRNA isotype between Ctrl and *Maf1* deficient mice as indicated. The regression line is colored, the x = y line is dotted.

In *Maf1* ^*−/−*^ mouse liver, Pol III occupancy was increased, and more so in the refed than in the fasted state (Bonhoure et al., 2020). Accordingly, we compared Pol III occupancy, as well as RNA and protein accumulation, in refed animals. We set up three cohorts of 13–14 week-old animals: Chow-fed *Maf1* ^*−/−*^ mice and both Chow- and HF-fed *Maf1* ^*hep−/−*^ mice, with Ctrl mice for each condition. Animals were weighed, fasted overnight, refed for 4 h, and sacrificed to provide samples for ChIPseq, RNAseq, and proteomic analyses (Supplementary Table S2). We first examined Pol III occupancy by ChIPseq using two to three replicates, each consisting of chromatin pools from two to three livers. We calculated Pol III occupancy for each cohort separately, considering the latest list of tRNA genes (GRCm38/mm10) (Chan and Lowe, 2016), as well as the genomic loci previously listed (Bonhoure et al., 2020). In each cohort, the mean and median scores were higher in mutant than in Ctrl mice (Figure 2A and Supplementary Table S3), as observed before in *Maf1* ^*−/−*^ mice under various conditions (Mange et al., 2017; Bonhoure et al., 2020).

A Limma analysis (Ritchie et al., 2015) identified differentially occupied genes (Supplementary Table S3), of which all were upregulated and nearly all were tRNA genes (see Legend to Figure 2B). The *Maf1* ^*−/−*^ cohort had the highest number of differentially occupied loci (142) followed by the HF-fed and Chow-fed *Maf1* ^*hep*−/−^ cohorts (91 and 72, respectively) (Figure 2B). Sixty tRNA genes, representing 21% of 287 tRNA genes occupied in at least one condition in one cohort, and one tRNA pseudogene were more occupied in all three cohorts. These 60 tRNA genes encompassed all isotypes except for Glu, Tyr, Cys and SeCys.

MvA plots comparing Chow-fed *Maf1* ^*−/−*^, Chow-fed *Maf1* ^*hep−/−*^, and HF-fed *Maf1* ^*hep−/−*^ to their respective Ctrl mice revealed increased occupancy of loci with low to medium occupancy levels, and smaller effects, especially in the Chow-fed *Maf1* ^*hep−/−*^ cohort, on highly occupied loci (Figure 2C). Indeed, the 60 loci more highly occupied in all datasets all correspond to tRNA genes (and one pseudo-tRNA gene) with low Pol III occupancy in Ctrl samples. This type of differential effect, which we have observed, albeit less pronounced, in previous experiments (Orioli et al., 2016; Mange et al., 2017), suggests that highly occupied loci have less margin for increased Pol III occupancy before reaching occupancy saturation.

To determine whether the absence of MAF1 had differential effects on different isotypes and isoacceptors, we calculated cumulative scores for all occupied tRNA genes and plotted them first by isotypes (Supplementary Table S4). As observed previously (Bonhoure et al., 2020), there was a progressive score increase in *Maf1* ^*−/−*^ livers with higher cumulated scores showing higher increases (Figure 2D). The same phenomenon was observed with the Chow-fed and HF-fed *Maf1* ^*hep−/−*^ cohorts (Figure 2D). Interestingly, there was a very good correlation between the number of genes in one isotype and cumulative Pol III occupancy scores (Supplementary Figure S2A). The same was true when relating gene number to total Pol III occupancy fold change (Supplementary Figure S2B), although some isotypes with large numbers of genes, in particular Glu, Arg, Leu, Ala, and Val, were consistently relatively distant from the regression line. This indicates smaller (Glu, Arg, Leu) or larger (Ala, Val) changes than could be expected just from the number of genes, consistent with the wide variation of occupancy scores for individual genes. Cumulative occupancy scores by isoacceptors (Supplementary Table S4) were also increased in the absence of *Maf1* in all three cohorts (Supplementary Figures S3A–C), and similar correlations with i) cumulative scores as well as ii) magnitude of score change were observed.

### 3.5 The absence of *Maf1* has little effect on liver mRNA accumulation

To assess mRNA accumulation, we collected liver RNA samples after refeeding (Supplementary Table S2) and subjected them to high throughput sequencing. A principal component analysis showed a good separation of Ctrl and *Maf1* ^*−/−*^ samples, but samples from both Chow- and HF-fed Ctrl and *Maf1* ^*hep−/−*^ mice were more intertwined (Supplementary Figure S4A). We determined differential expression for each cohort with the R package Limma, and used cutoffs on both adjusted *p*-values (<0.05) and fold change (|log2(FC)|>1) to select candidates for examination (Supplementary Table S5).

**FIGURE 3:**
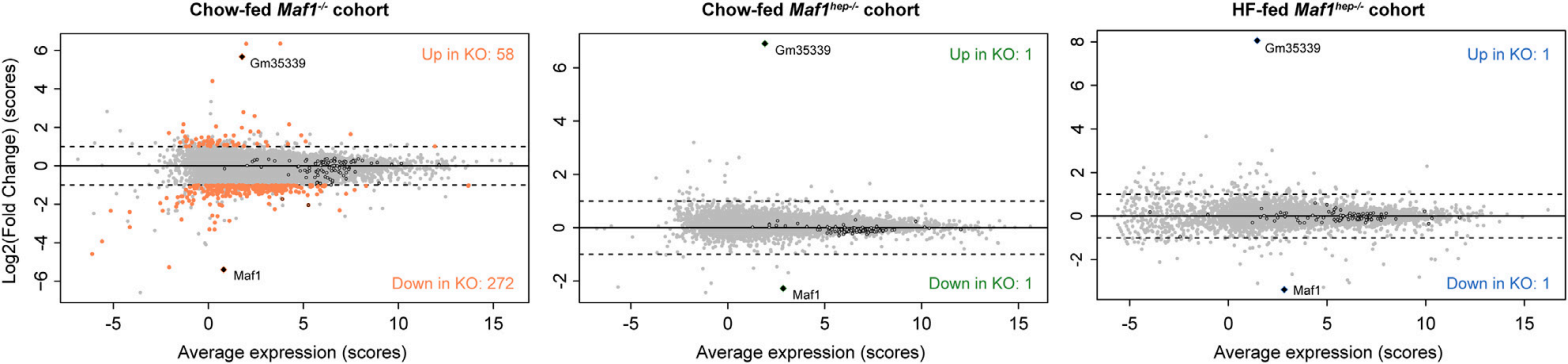
MAF1 absence has little effect on accumulation of mRNAs. MvA plots showing average mRNA accumulation scores ((Ctrl scores + *Maf1* knockout score)/2) in the indicated cohorts on the *X*-axes and the log2(FC) on the *Y*-axes. Grey dots: mRNAs that do not change significantly; colored dots: mRNAs showing significant changes; black circles: TOP mRNAs. Diamonds: Maf1 and Gm35339 RNAs.

We previously reported RNAseq results for the Chow-fed *Maf1* ^*−/−*^ mice using less stringent cutoffs (Bonhoure et al., 2020). The present analysis of the same data identified 330 differentially expressed mRNAs (out of 12992 sequenced), with the large majority (272) being, as before, decreased (Supplementary Table S5, Figure 3). Strikingly, the Chow or HF-fed *Maf1* ^*hep−/−*^ cohorts revealed no changing RNAs except for *Maf1* and *Gm35339* (Figure 3 and Supplementary Table S5), the locus downstream of *Maf1* whose predicted WD40 repeat protein was undetectable (see below) and whose RNA upregulation does not seem to contribute to the *Maf1* ^*−/−*^ mouse phenotype (Bonhoure et al., 2015). Moreover, the slight decrease in TOP mRNAs reported previously in *Maf1* ^*−/−*^ mice was virtually absent in the two other cohorts (Figure 3 and Supplementary Figure S4B). With much less stringent cutoffs (adjusted *p*-value <0.1 and fold change |log2(FC)|>0.5), only two additional differences were found (*Sclo1a1* and *Cdkn1c*), in the HF-fed *Maf1* ^*hep−/−*^ cohort. These results are in line with our previous suggestion that MAF1 does not act by directly impacting Pol II transcription (Bonhoure et al., 2015; Orioli et al., 2016). Rather, any effect on mRNA accumulation likely results from changes in Pol III transcription. Moreover, the variations in mRNA accumulation observed in the liver of *Maf1* ^*−/−*^ mice probably reflect systemic, indirect effects linked to the absence of MAF1 in the entire body and throughout the entire life of the mouse, including fetal development.

### 3.6 Metabolic changes in the liver are dampened in *Maf1* ^*hep−/−*^ mice

The concept that futile tRNA synthesis and turnover underlies the metabolic inefficiency and lean phenotype of *Maf1* ^*−/−*^ mice implies that metabolic reprograming in these animals is a cell intrinsic property; it is expected to occur in all tissues given the ubiquitous expression of tRNA genes and MAF1 (Bonhoure et al., 2015; Willis et al., 2018). To examine this, we profiled metabolites in central metabolic pathways of Chow-refed *Maf1* ^*hep−/−*^ mice using the same quantitative platform employed to investigate liver metabolism in refed *Maf1* ^*−/−*^ mice. A comparative analysis revealed far fewer metabolites with significant changes in *Maf1* ^*hep−/−*^ *versus Maf1* ^*−/−*^ mice (7 *versus* 43 metabolites, Supplementary Table S6). However, twenty additional metabolites showed a trend towards differential abundance at a relaxed stringency (*p*-value < 0.1), with most showing fold changes smaller but in the same direction as in *Maf1* ^*−/−*^ mice, pointing to effects on the same pathways. For example, amino acids and related metabolites were generally elevated, as was urea, consistent with *Maf1* ^*−/−*^ mice where increased autophagy and amino acid deamination (needed for carbon skeleton entry into the TCA cycle) support their increased energy demand. Other shared changes involve altered cellular methylation potential (decreased SAM/SAH ratio) and effects on choline demethylation (betaine, dimethylglycine and sarcosine) and polyamine (putrescine and spermidine) pathways. Thus, the observations that i) the metabolites affected in *Maf1* ^*hep−/−*^ mice are largely a subset of the ones changed in *Maf1* ^*−/−*^ mice and ii) the changes are in the same direction in both genotypes support the idea that deletion of *Maf1* has a cell autonomous effect on metabolism. In addition, however, the higher number of metabolites affected and the larger magnitude of changes in *Maf1* ^*−/−*^ mice reveal a large systemic contribution of *Maf1* loss in the whole animal on metabolism in the liver.

### 3.7 Differences in protein accumulation in *Maf1* mutant mice

**FIGURE 4:**
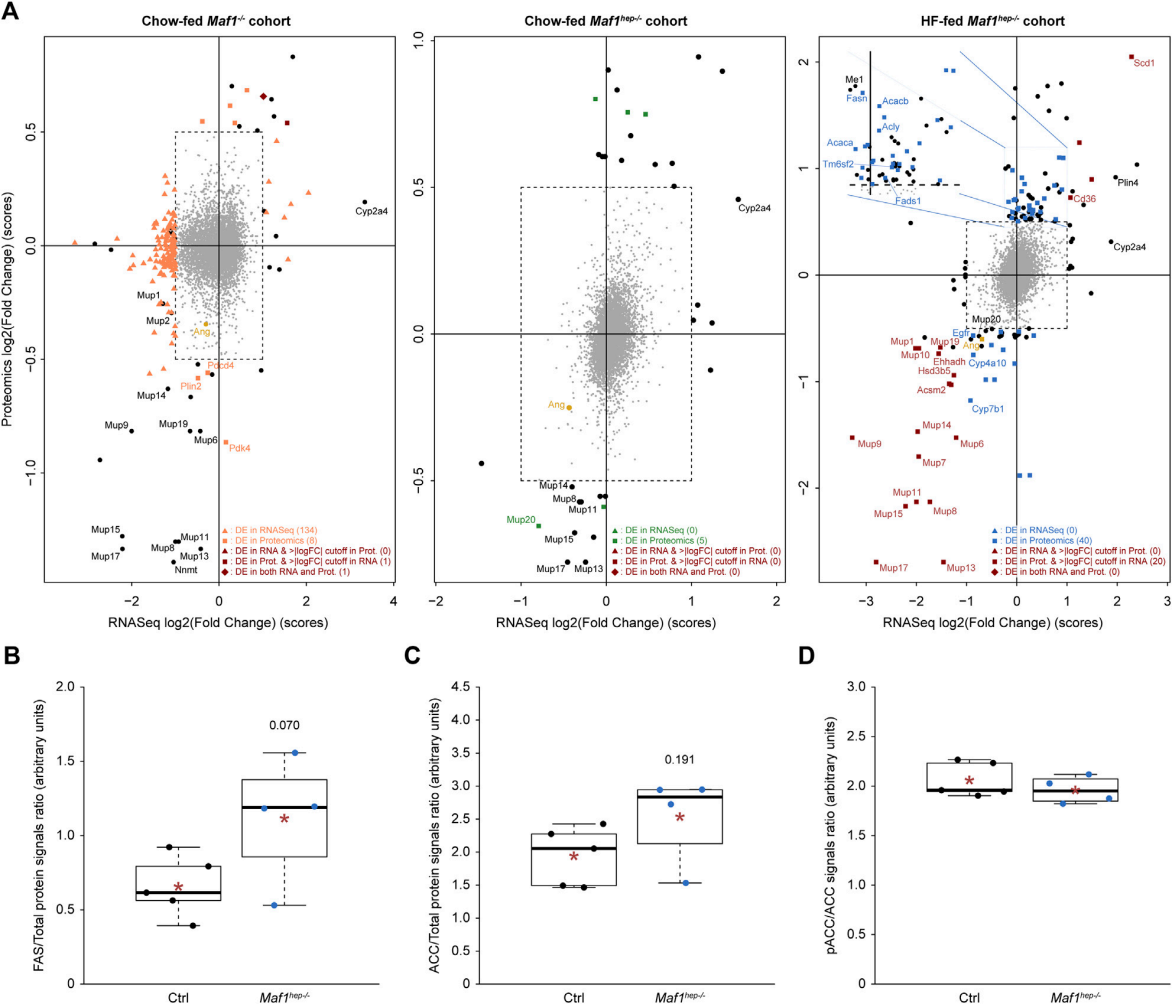
Differentially accumulated proteins and mRNA in the three cohorts. **(A)** The *x*-axis indicates mRNA accumulation log2(FC), the *y*-axis protein accumulation log2(FC). The central square delimits log2(FC) and *p*-values below cutoffs for both mRNA and protein. Orange (first panel), green (second panel) and blue (third panel) triangles: mRNAs passing cutoffs (adjusted *p*-values<0.05 and |log2(FC)|>1). Squares of the same colors: proteins passing cutoffs (*p*-values<0.05 and |log2(FC)|>0.5). Dark brown squares: factors passing the log2(FC) cutoff for both mRNA and protein but *p*-value cutoff only for proteins. Dark brown diamonds: factors passing both cutoffs for both mRNA and protein. There were no factors passing the log2(FC) cutoff for both mRNA and protein but the *p*-value cutoff only for mRNA. Black circles outside of the central dotted square: mRNAs or proteins passing only one or the two log2(FC) cutoffs, but no *p*-value cutoff. Numbers in parenthesis following the legends in the bottom right of each panel: number of factors satisfying the indicated conditions. For optimal presentation, Gm20547 and Gm20671, with the largest differential mRNA accumulation between Chow-fed *Maf1* ^*−/−*^ and Ctrl (log2(FC) = −6.591 and −4.583, respectively), and Ppp1r13b, with the largest differential protein accumulation between Chow-fed *Maf1* ^*hep−/−*^ and Ctrl (log2(FC) = 3.636), are not indicated in the relevant panels. Note also that the value attributed to MUP1 is that of the lower (MUP1/10) of the two MUP1-containing duplets (MUP1/10 and MUP1/15). **(B**–**D)** WES™ quantifications of FAS **(B)**, total ACC **(C)** and the inactive phosphoACC over total ACC **(D)** proteins in the HF-fed *Maf1* ^*hep−/−*^ cohort. Ctrl *n* = 5 and *Maf1* ^*hep−/−*^ *n* = 4. The boxes encompass upper and lower quartile, the whiskers extend to 1.5 times the interquartile range of the box. Red star, mean; horizontal bar, median. Numbers inside figures indicate *p*-values.

Protein extracts were analyzed by mass-spectrometry using tandem mass tags and four or five biological replicates per condition (Supplementary Table S2). MAF1 was not detected in Ctrl mice, perhaps due to its low abundance and small size, nor was Uniprot A0A140LHH9, encoded by *Gm35339,* despite its predicted large size, unique sequence, and presence in the reference database. To focus on proteins likely to be synthesized endogenously in the liver and to compare variations at both RNA and protein levels, we selected proteins with a corresponding mRNA in the RNAseq data (6,360–6,544 proteins depending on the cohort) and computed differential accumulation. Cutoffs of adjusted *p*-values <0.05 and fold change (|log2(FC)|>0.5) showed no significant changes. To avoid loss of true positives (Pascovici et al., 2016), we used *p*-values (rather than adjusted *p*-values) <0.05 (Supplementary Table S7), revealing 9, 5, and 56-60 proteins differentially expressed in the *Maf1* ^*−/−*^, Chow-fed *Maf1* ^*hep−/−*^, and HF-fed *Maf1* ^*hep−/−*^ cohorts, respectively (see Figure 4A, squares). A gene ontology analysis of biological process terms for differentially expressed proteins in the HF-fed *Maf1* ^*hep−/−*^ mice identified prominently lipid and sterol metabolism, nuclear receptors and DNA replication (Supplementary Table S8). We focused on these mice to group proteins and mRNAs according to these and other processes, and expanded the analyses with the few changes observed in the *Maf1* ^*−/−*^ and Chow-fed *Maf1* ^*hep−/−*^ mice when relevant.

#### 3.7.1 Proteins changes involving the synthesis, catabolism, export and storage of fatty acids

**FIGURE 5:**
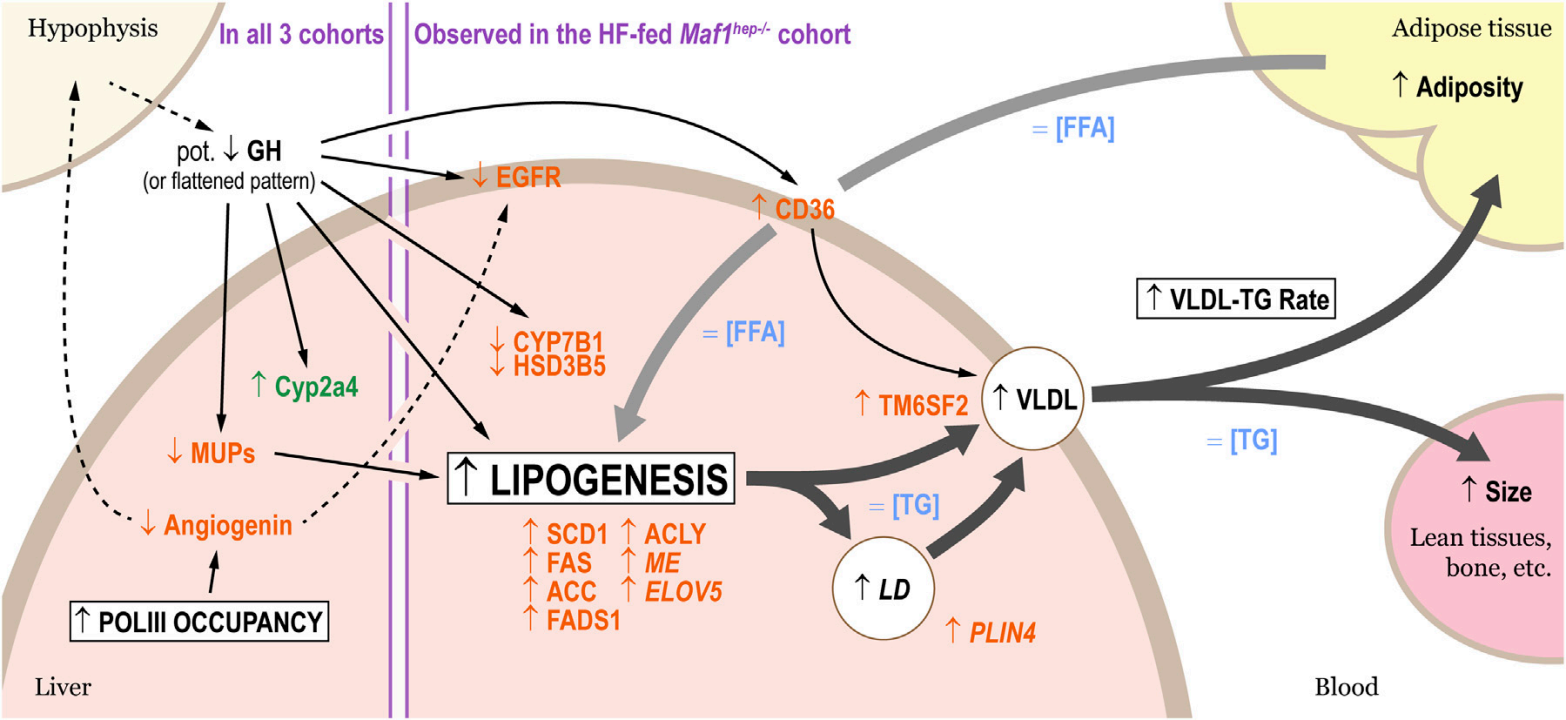
Altered lipid metabolism in HF-fed *Maf1* ^*hep−/−*^ mice. Four main processes are affected: Pol III occupancy, lipogenesis, accumulation of lipids in lipid droplets, and VLDL release rate. Proteins whose levels were altered are indicated in orange, mRNAs in green. Changes in serum factors or hepatic concentrations are indicated in blue. The broad arrows designate lipids fluxes (black: from the liver; grey: into the liver), the thin arrows known (solid lines) or hypothesized (dotted lines) regulation processes. Terms in italics indicate tendencies, and “Pot.” stands for “potential.”

The lipid metabolism gene ontology terms (Supplementary Table S8) included mostly upregulated proteins (Figure 4A, third panel; Figure 5 and Supplementary Table S7), several of which are involved in important rate-limiting steps in fatty acid synthesis (Saponaro et al., 2015). Among these are: ATP citrate lyase (ACLY), Acetyl-CoA carboxylase (ACC1 and ACC2), fatty acid synthase (FAS), malic enzyme (with a log2(FC) of 1.02 but a *p*-value of 0.083), the fatty acid elongase ELOV5 (*p*-value of 0.028 but log2(FC) of 0.491), and fatty acid desaturases (SCD1 and FADS1). In addition, the Perilipin PLIN4 (log2(FC) of 0.918 but *p*-value of 0.054), seemed upregulated, consistent with the tendency to increased lipid droplets (see above Figures 1E, F) (Itabe et al., 2017; Sztalryd and Brasaemle, 2017). ACSM2, LBP (*Ehhadh* gene), and CYP4A10 were downregulated, perhaps contributing to a slight decrease in fatty acid catabolism.

To confirm upregulation of lipogenesis, we analyzed FAS and ACC1/2 in the HF-fed mice by capillary-based Western blot (WES™). Both FAS and ACC levels were modestly increased relative to Ctrl with *p*-values of 0.07 and 0.191, respectively [Figures 4B, C: note that in both cases, the very low outlier values for FAS and ACC in the *Maf1* ^*hep−/−*^ group originate from a single underweight mouse (Supplementary Table S2), without which the *p*-values would be 0.005 and 0.007, respectively]. The ratio of inactive serine 79-phosphorylated ACC to total ACC was similar in Ctrl and *Maf1* ^*hep−/−*^ mice (Figure 4D), indicating similar specific activity. These results indicate an overall higher rate of lipogenesis in the liver of HF-fed *Maf1* ^*hep−/−*^ mice, with higher production of mono- and polyunsaturated fatty acids. Importantly, we previously also observed higher levels of *de novo* lipogenesis in *Maf1* ^*−/−*^ mice by measuring D2O incorporation into liver triglyceride (Bonhoure et al., 2015).

Also upregulated were CD36 and TM6SF2 (Figure 4A). CD36 mediates fatty acid uptake (used for lipogenesis), functions as a metabolic sensor and, in the liver, is increased by a HF diet (He et al., 2011). Its total or hepatic-specific knockout lowers VLDL-secretion (Nassir et al., 2013; Wilson et al., 2016), whereas its hepatic-overexpression under a HF diet increases VLDL-secretion (Garbacz et al., 2016). TM6SF2, a transmembrane protein highly expressed in liver, promotes accumulation of neutral lipids in VLDL (Li et al., 2018). The over-accumulation of these two proteins is thus consistent with increased VLDL secretion and increased lipogenesis in HF-fed *Maf1* ^*hep−/−*^ mice (Figure 5).

In contrast to HF-fed *Maf1* ^*hep−/−*^ mice, only three proteins with important roles in metabolism were downregulated in the liver of Chow-fed *Maf1* ^*−/−*^ mice (Figure 4A, first panel, and Supplementary Table S7): PDK4, a pyruvate dehydrogenase inhibitory kinase (Park et al., 2018), whose reduction in *Maf1* ^*−/−*^ mice adds to the previously observed reduced phosphorylation of the pyruvate dehydrogenase E1a subunit that drives higher Acetyl-CoA production in line with the higher energetic demands of these mice (Bonhoure et al., 2015; Willis et al., 2018); Perilipin-2 (PLIN2), whose decreased levels on the surface of lipid droplets allows access to lipases (Sztalryd and Brasaemle, 2017), consistent with lower lipid droplet numbers and decreased TG storage in the liver of *Maf1* ^*−/−*^ mice (Bonhoure et al., 2015); and PDCD4, whose knockout in HF-fed mice diminishes hepatic steatosis and TG serum levels and promotes a lean phenotype (Wang et al., 2013), consistent with the lean phenotype and absence of hepatic steatosis in *Maf1* ^*−/−*^ mice. Decreased levels of PDK4, PLIN2, and PDCD4 are thus consistent with the phenotype of *Maf1* ^*−/−*^ mice (Bonhoure et al., 2015). Also consistent is the decrease of NNMT (*p*-value of 0.061), as previously reported (Bonhoure et al., 2015), whose deregulation is associated with obesity resistance through modulation of NAD+ levels (Kraus et al., 2014).

#### 3.7.2 Decreased Major Urinary Proteins

The 22 murine *Mup* genes are clustered on chromosome 4 and encode highly homologous Major Urinary Proteins (MUPs) (Zhou and Rui, 2010; Charkoftaki et al., 2019). Despite their high homology, the proteomics analysis unambiguously identified MUP2, 3, 7, 14, 20 and 21, and, in the HF-fed cohort, MUP19. Additional MUPs were identified by peptides specific to both MUP8 and 11, MUP13 and 17, MUP1 and 10, MUP1 and 15 in the three cohorts, MUP6 and 9 in the HF-fed cohort, and MUP6, 9 and 19 in the two Chow-fed cohorts. Thus, among the 56 to 60 proteins differentially expressed in the HF-fed *Maf1* ^*hep−/−*^ cohort, 8 to 12 MUPs (MUP7, 14, 19, MUP1/10, MUP1/15, MUP6/9, MUP8/11 and MUP13/17) were downregulated (Figure 4A; Figure 5). The other three detected members of this family (MUP2, MUP3 and MUP20) were also downregulated, albeit with one value just missing one of the two cutoffs (Supplementary Table S7). Moreover, several MUPs were downregulated in the *Maf1* ^*−/−*^ and Chow-fed *Maf1* ^*hep−/−*^ cohorts (Figure 4A).

MUP levels are regulated by Growth Hormone (GH). Interestingly, EGFR, CYP7B1 and HSD3B5, similarly regulated by GH (Baik et al., 2011), were also decreased, and CD36, which can be negatively regulated by GH, was increased (Figure 4A; Figure 5). In these cases, the corresponding mRNAs were also affected, albeit at levels that did not pass our cutoffs (Figure 4A).

#### 3.7.3 Decreased accumulation of Angiogenin

Angiogenin, a ribonuclease mainly expressed in, and secreted by, the liver (Lyons et al., 2017), was significantly decreased in HF-fed *Maf1* ^*hep−/−*^ mice (Figure 4A). Angiogenin owes its name to its promoting angiogenesis during tumor growth but has numerous other functions such as i) promoting the production of the large ribosomal RNAs (Pizzo et al., 2013; Lyons et al., 2017), ii) cleaving tRNAs under stress conditions thus generating tRNA halves that can inhibit translation and promote survival through inhibition of apoptosis (Sheng and Xu, 2016), and iii) other ill-defined activities (Lyons et al., 2017; Rashad et al., 2020). Notably, the EGFR decrease in HF-fed *Maf1* ^*hep−/−*^ mice (Figure 4A; Figure 5) mentioned above may be linked to the Angiogenin decrease, since Angiogenin was recently identified as a potent EGFR ligand in humans (Wang et al., 2018; Wang et al., 2019).

#### 3.7.4 Common features among cohorts

To identify common trends in the three cohorts, we first identified proteins or mRNAs passing our cutoffs on log2(FC) only, but in all three cohorts. We found four to seven MUP proteins (MUP1/15, 8/11, 13/17 and 14), all downregulated, and one upregulated mRNA, *Cyp2a4* (Figure 4A). CYP2A4 is highly expressed in the liver, in females more than in males where its expression is repressed by GH (Aida and Negishi, 1993; Sueyoshi et al., 1999).

Second, we examined smaller but consistent changes (|log2(FC)|>0,25, *p*-value<0.1) occurring in at least two cohorts. With these criteria, we identified only 20–23 proteins (Supplementary Table S9) and no mRNAs. Thirteen to fourteen of these were in the Chow-fed and HF-fed *Maf1* ^*hep−/−*^ cohorts. They include ELOV5 and TM6SF2, which as described above function in elongation of essential fatty acids and neutral lipid loading of VLDL, and two to three MUPs (MUP1/10 and 20). Collectively, these changes indicate a slight lipid dysregulation in the Chow-fed *Maf1* ^*hep−/−*^ cohort, consistent with the weight increase at old age (Figures 1B, C). Five to seven proteins including one to three MUPs (MUP6/9/19) were affected in the Chow-fed *Maf1* ^*−/−*^ and HF-fed *Maf1* ^*hep−/−*^ cohorts.

A single protein, RNase 4, was downregulated in both the Chow-fed *Maf1* ^*−/−*^ and *Maf1* ^*hep−/−*^ cohorts, and a single protein, Angiogenin, was downregulated in all three cohorts. Angiogenin and RNase 4 are encoded by a shared transcription unit (Dyer and Rosenberg, 2005), whose promoter contains two tRNA genes. In reporter assays, deletion of these tRNA genes increased transcription, suggesting that they dampen Pol II transcription from the promoter (Sheng et al., 2014). Indeed, the two tRNAs (chr14_293_tRNATyr_GTA+ and chr14_294_tRNAPro_TGG+) are slightly more occupied by Pol III in the absence of MAF1 (Supplementary Table S3), suggesting a direct effect on Angiogenin and, in two out of three cohorts, RNase 4, downregulation.

## 4 Discussion

### 4.1 Contrasting phenotypes of *Maf1* ^*−/−*^ and *Maf1* ^*hep−/−*^ mice

*Maf1* ^*hep−/−*^ mice, in which the *Maf1* gene is excised specifically in hepatocytes in the late fetal to neonatal stages are fundamentally different from *Maf1* ^*−/−*^ mice, which are born from mothers lacking *Maf1* and which themselves lack *Maf1* in every organ during their entire development. Indeed, these mice have different phenotypes. Whereas *Maf1* ^*−/−*^ mice are smaller, lighter and leaner than Ctrl counterparts, displaying increased overall energy expenditure (Bonhoure et al., 2015), the Chow-fed *Maf1* ^*hep−/−*^ have the same weight as Ctrl mice except in old age, when they tend to become slightly heavier. Under a HF diet, *Maf1* ^*hep−/−*^ mice are clearly larger, heavier, and fattier than Ctrl mice, with dysregulation of hepatic lipid accumulation and increased VLDL-TG release.

Proteomic analysis revealed changes mostly in the HF-fed *Maf1* ^*hep−/−*^ cohort, where they indicated i) increased lipogenesis, ii) increased VLDL release, and iii) a tendency to increased liver lipid droplets (Figure 5). More generally, this is consistent with the increased size, weight and fat content of these animals. The increased lipogenesis and adiposity of *Maf1* ^*hep−/−*^ mice parallels findings in the liver of *Maf1* ^*−/−*^ mice, where measurements of D2O incorporation into TG revealed increased *de novo* lipogenesis (Bonhoure et al., 2015), and in *C. elegans* and mammalian cell lines with reduced MAF1 levels (Khanna et al., 2014; Palian et al., 2014). The lipogenesis effect is thus shared in these different models but leads to different adiposities, probably reflecting an offset of energy supply by energy demand in some but not all systems. In *Maf1* ^*−/−*^ mice, the lean phenotype can be explained by the findings that i) mammalian MAF1 is a chronic repressor of Pol III transcription (Orioli et al., 2016; Bonhoure et al., 2020), and ii) the absence of MAF1 drives a futile tRNA synthesis and degradation cycle (Bonhoure et al., 2015; Willis et al., 2018). Notably, in these *Maf1* ^*−/−*^ mice the futile cycle is most pronounced in white adipose tissue, spleen, brown adipose tissue, brain, heart, small intestine and kidney, with liver and quadriceps displaying the smallest effect as determined by precursor tRNA ratios (Bonhoure et al., 2015; Bonhoure et al., 2020). It seems likely, therefore, that in mice lacking MAF1 only in hepatocytes, the increased energy demand resulting from futile tRNA synthesis and degradation only in the liver does not exceed energy supply. This may also be the case in *C. elegans* and mammalian cell lines, where in addition, the knockdown of MAF1 may have been incomplete.

Thus, the proteomic analysis reveals altered levels of only a few proteins, which over the life of the animal appear to have an effect on total weight and size via increased fatty acid synthesis and lipogenesis. Metabolic changes in the livers of both *Maf1* ^*−/−*^ and *Maf1* ^*hep−/−*^ mice might also occur through changes in pathway flux due to altered levels of metabolites (Bonhoure et al., 2015) (Supplementary Table S6) and posttranslational mechanisms.

The striking differences in the overall phenotypes of *Maf1* ^*−/−*^ and *Maf1* ^*hep−/−*^ mice (lean *versus* fat) illustrates the interplay between different tissues of an organism. Another example is the reported effect of MAF1 on bone mass (Phillips et al., 2022). Primary bone marrow cells isolated from *Maf1* ^*−/−*^ mice showed reduced osteoblastogenesis *ex vivo* and mice overexpressing MAF1 in cells from the mesenchymal lineage had increased bone mass (Phillips et al., 2022). In contrast, *Maf1* ^*−/−*^ mice themselves had a high bone mass phenotype, indicating that the absence of MAF1 during development and/or in all organs leads to different effects than the absence of MAF1 in a specific cell type.

### 4.2 Pol III occupancy and translation

Pol III occupancy was increased in both *Maf1* ^*−/−*^ and *Maf1* ^*hep−/−*^ livers, consistent with previous observations under different conditions in mouse liver and in cell lines (Orioli et al., 2016; Mange et al., 2017; Bonhoure et al., 2020). Loci with increased Pol III occupancy encompassed tRNA genes of most isotypes and isoacceptors. Moreover, the magnitude of the increase correlated with the number of tRNA genes for each isotype or isoacceptor. Thus, the expansion of isotype or isoacceptor tRNA genes allows for a greater amplitude of expression, something especially relevant for Pol III genes, whose small size restricts polymerase occupancy of the transcription unit. Importantly, however, we have shown previously that increased occupancy and transcription of tRNA genes leads to increased pre-tRNA synthesis but has little or no effect on mature tRNAs levels (Bonhoure et al., 2015; Bonhoure et al., 2020). Indeed, the only translation effect observed in the liver of *Maf1* ^*−/−*^ mice was a small reduction (rather than a perhaps expected increase) in global translation (Bonhoure et al., 2020). This may have resulted from a systemic, non-cell-autonomous effect since it was not observed in *Maf1* ^*hep−/−*^ livers as determined by puromycin incorporation (Supplementary Figure S5). Thus, in both *Maf1* ^*−/−*^ and *Maf1* ^*hep−/−*^ mice, any putative effect on translation resulting directly from the absence of MAF1 would be specific to certain mRNAs rather than global.

### 4.3 Is MAF1 directly affecting Pol II transcription?

In *C. elegans*, reducing the level of MAF1 resulted in increased transcription of genes involved in lipid transport and synthesis, specifically *vit*/Vitellogenins, *pod-2*/ACC1 and fasn-1/FASN (Khanna et al., 2014). Increased expression of *Fasn* and *Acc1* mRNAs was also seen in mouse hepatoma cells following MAF1 knockdown and was attributed to decreased direct recruitment of MAF1 to the corresponding promoters (Palian et al., 2014). Moreover, CREB-targeted genes were recently identified as enriched for MAF1 binding (Tsang et al., 2023). These and other findings suggesting that MAF1 can function as a typical transcription factor, binding to, and thereby activating or repressing, certain Pol II promoters are difficult to reconcile with i) the absence of a DNA-binding motif in the protein, ii) genome-wide ChIPseq studies detecting little or no MAF1 binding to Pol II promoters, including the Fasn promoter (Orioli et al., 2016), and iii) the present RNAseq analysis which revealed few changes in the *Maf1* ^*hep−/−*^ cohorts. This last point suggests that the RNAseq changes seen in the *Maf1* ^*−/−*^ cohort (Bonhoure et al., 2020) resulted largely from a systemic effect rather than a MAF1-specific effect in the liver.

Changes in Pol III transcription can clearly, however, affect expression of Pol II genes. For example, reducing Pol III transcription with the chemical inhibitor ML-60218 or reducing the levels of BRF1, a Pol III transcription factor required by most Pol III genes, enhanced expression of pro-adipogenic genes, while reducing MAF1 levels had the opposite effect (Chen et al., 2018). Altered patterns of Pol II gene expression following perturbations in Pol III transcription can be complex: In ST2 cells, *Maf1* overexpression, *Brf1* knockdown, and ML-60218 treatment each resulted in gene expression alterations as determined by RNAseq, but with very little overlap between these conditions (Phillips et al., 2022). This likely reflects unique effects of the various treatments. For example, MAF1 does not inhibit all Pol III genes equally (Orioli et al., 2016; Yeganeh et al., 2017) and a BRF1 decrease might not affect BRF2-dependent Pol III genes. Further, if ML-60218 treatment inhibits Pol III elongation on the DNA as α-amanitin does for Pol II, a blocked Pol III enzyme may affect transcription of Pol II genes overlapping or containing Pol III genes. More generally, down or upregulation of Pol III genes can impact Pol II gene expression by transcriptional interference (Hull et al., 1994; Korde et al., 2014; Yeganeh et al., 2017), or by affecting processes involving Pol III RNA products such as Pol II transcription elongation, pre-mRNA and rRNA processing, translation, DNA replication, and, in particular for tRNA halves and Pol III-produced micro-RNAs, other regulatory processes that remain to be fully determined (Canella et al., 2010; Policarpi et al., 2017).

### 4.4 Is *Maf1* impacting Growth Hormone and Angiogenin levels?

To uncover effects that might link both *Maf1* ^*−/−*^ and *Maf1* ^*hep−/−*^ phenotypes with the known biochemical function of MAF1 in Pol III transcription repression, we examined common changes. Interestingly, several changes point to an alteration of GH regulation (Figure 5). First, the decrease of many MUPs, not only in the HF-fed *Maf1* ^*hep−/−*^ cohort but for four to seven of them, in all three cohorts (Figure 4A). MUPs are mainly expressed in adult liver and are tightly regulated by pituitary hormones, in particular GH (Holloway et al., 2006; Fan et al., 2014; Connerney et al., 2017; Lau-Corona et al., 2017; Penn et al., 2022), their urinary release being in fact used as a proxy for measuring male-specific pulsatile GH release (Knopf et al., 1983; Shaw et al., 1983). They bind and transport small hydrophobic molecules such as steroid hormones, retinoids, lipids, and odorants, and in urine are involved in pheromone communication. They are also implicated in regulating energy expenditure. Thus, in obese db/db mice and in wild-type mice fed a HF diet, MUP1 mRNA and protein are decreased (Hui et al., 2009; Zhou et al., 2009; Chen et al., 2015). Hepatic-specific *Mup1* overexpression decreased hepatic TG, *Fasn* and *Scd1* mRNA levels, and liver weight (Zhou et al., 2009), whereas chronic administration of recombinant MUP1 increased energy expenditure and locomotor activity (Hui et al., 2009). MUP1 thus appears to negatively regulate lipogenesis. This is consistent with the increased adiposity and hepatic lipid biosynthesis in HF-fed *Maf1* ^*hep−/−*^ mice, albeit not with the decreased adiposity of Chow-fed *Maf1* ^*−/−*^ mice, where many other factors resulting from the absence of MAF1 in all organs are, however, at play. Second, we observed an increase of female biased *Cyp2a4* mRNA in all three cohorts, and a decrease of male biased CYP7B1 and HSD3B5 proteins in HF-fed *Maf1* ^*hep−/−*^ mice (Figure 4A). These observations mirror reduced hepatic *Cyp7b1* mRNA upon pituitary gland ablation, and increased *Cyp2a4* mRNA in males whose GH plasma profile is modified to mimic that of females (Holloway et al., 2006; Jarukamjorn et al., 2006; Connerney et al., 2017; Lau-Corona et al., 2017). Third, we observed decreased EGFR in HF-fed *Maf1* ^*hep−/−*^ mice, which may seem surprising as EGFR promotes cell proliferation, and liver weights of HF-fed *Maf1* ^*hep−/−*^ seemed to increase in the refed state, albeit the Bonferroni-corrected *p*-value was above our threshold (Supplementary Table S1). The EGFR decrease can however be compensated by Hepatocyte Growth Factor (HGF) acting via its receptor HGFR (Bhushan and Michalopoulos, 2020). At any rate, *Egfr* transcription and protein levels are regulated by GH (Gonzalez et al., 2021). Thus, all these effects are consistent with a slightly decreased overall level of plasma GH and/or a “feminization” of GH release by the pituitary gland, i.e., a change in the pulsatile male pattern of GH secretion towards a more constant and uniform secretion pattern, in line with the increased lipogenesis observed in the HF-fed *Maf1* ^*hep−/−*^ mice. It is worth noting that GH is implicated in liver lipogenesis and hepatosteatosis (Takahashi, 2017): changes in GH secretion might thus be at least partially responsible for the increased hepatosteatosis phenotype observed in the HF-fed *Maf1* ^*hep−/−*^ mice (Barclay et al., 2011; Le Magueresse-Battistoni, 2021).

Another intriguing observation is the Angiogenin decrease in all three cohorts, the one and only protein, even with the relaxed cutoffs, to do so. RNase 4, which is expressed from the same gene (but can be differentially expressed through a CTCF-dependent regulatory mechanism) (Sheng et al., 2014), was diminished in two of the three cohorts. These observations can be directly linked to the absence of MAF1: The main Angiogenin (and RNase 4) promoter is downregulated by two tRNA genes located just downstream (Sheng et al., 2014), in line with observations that a highly transcribed Pol III transcription unit located inside a Pol II gene can downregulate expression of the Pol II gene by transcription interference (Korde et al., 2014; Yeganeh et al., 2017). In the present study, in the refed state, we find these two tRNA genes (gold labeled in Figure 2C) already highly occupied in the Ctrl mice, and thus only slightly more occupied in the absence of MAF1, and although Angiogenin mRNA is decreased, the effect is too small to pass our stringent cutoff in the RNAseq study [log2(FC)/adj. *p*-values: Chow-fed *Maf1* ^*−/−*^, −0.301/0.249; Chow-fed *Maf* ^*-hep−/−*^, −0.435/0.867; HF-fed *Maf* ^*-hep−/−*^, −0.693/0.281]. However, previous studies show that Pol III occupancy of these tRNA genes can vary, and in some cases point to an anti-correlation between Pol II and Pol III occupancy. Thus, they are clearly differentially occupied in *Maf1* ^*−/−*^ and Ctrl mice in fasted or refed states (Bonhoure et al., 2020), or during the diurnal cycle (Mange et al., 2017), as well as in a human cell line transfected with si*Maf1* (Orioli et al., 2016). Moreover, low Pol III occupancy of the two tRNA genes and high Pol II occupancy of the Ang gene was observed 48 h after hepatectomy, a situation that was reversed 60 h after hepatectomy (Yeganeh et al., 2017). Angiogenin (albeit as a tendency) and RNAse 4 mRNA were both downregulated in differentiated osteoblasts treated with sh*Maf1* (Phillips et al., 2022), as determined by RNAseq. And in cells treated with a shRNA against *Brf1*, both mRNAs were slightly upregulated (Phillips et al., 2022). Confusingly, however, chemical inhibition of Pol III transcription with ML-60218 led to downregulation of Angiogenin mRNA (Phillips et al., 2022), a result that would however be explained if ML-60218 blocked Pol III on the template, as α-amanitin does for Pol II.

Downregulation of Angiogenin is intriguing for several reasons. Angiogenin is a secreted factor readily taken up by cells through a receptor-mediated mechanism. It is expressed in many organs and plays various key roles (Garnett and Raines, 2022), such as promoting axonal growth during early brain development (Subramanian and Feng, 2007) or formation of blood vessels during embryogenesis. It could thus have very different non-cell-autonomous effects in different genotypes such as *Maf1* ^*−/−*^ and *Maf1* ^*hep−/−*^ mice.

Secreted Angiogenin and GH levels might be somehow linked, as i) patients with increased or decreased GH levels displayed correspondingly increased or decreased circulating Angiogenin levels, and ii) a significant correlation was observed between plasma levels of Angiogenin and of insulin-like growth factor (IGF-1), the main effector of GH, in both patients and healthy subjects (Silha et al., 2005). If Angiogenin were indeed linked with GH or IGF-1, it could explain how the localized effect of lowered Angiogenin in *Maf1* ^*hep−/−*^ hepatocytes, the main site of Angiogenin production, might affect hypophysis release of GH. Future work should assess any possible role of Angiogenin in mediating the effects triggered by a lack of MAF1.

## Data Availability

The sequencing data are accessible in the NCBI Gene Expression Omnibus (GEO; https://www.ncbi.nlm.nih.gov/geo/) under accession number GSE224917. The MS data together with MaxQuant output tables are available via the ProteomeXchange data repository (www.proteomexchange.org) with the dataset identifier PXD040086.
