# Supplementary material for: Contrasting effects of whole-body and hepatocyte-specific deletion of the RNA polymerase III repressor Maf1 in the mouse
Source: Front Mol Biosci. 2023 Dec 5;10:1297800. doi: 10.3389/fmolb.2023.1297800 (PMC10746880; doi:10.3389/fmolb.2023.1297800)
Supplement: Supplementary file 5 [file DataSheet1.pdf]

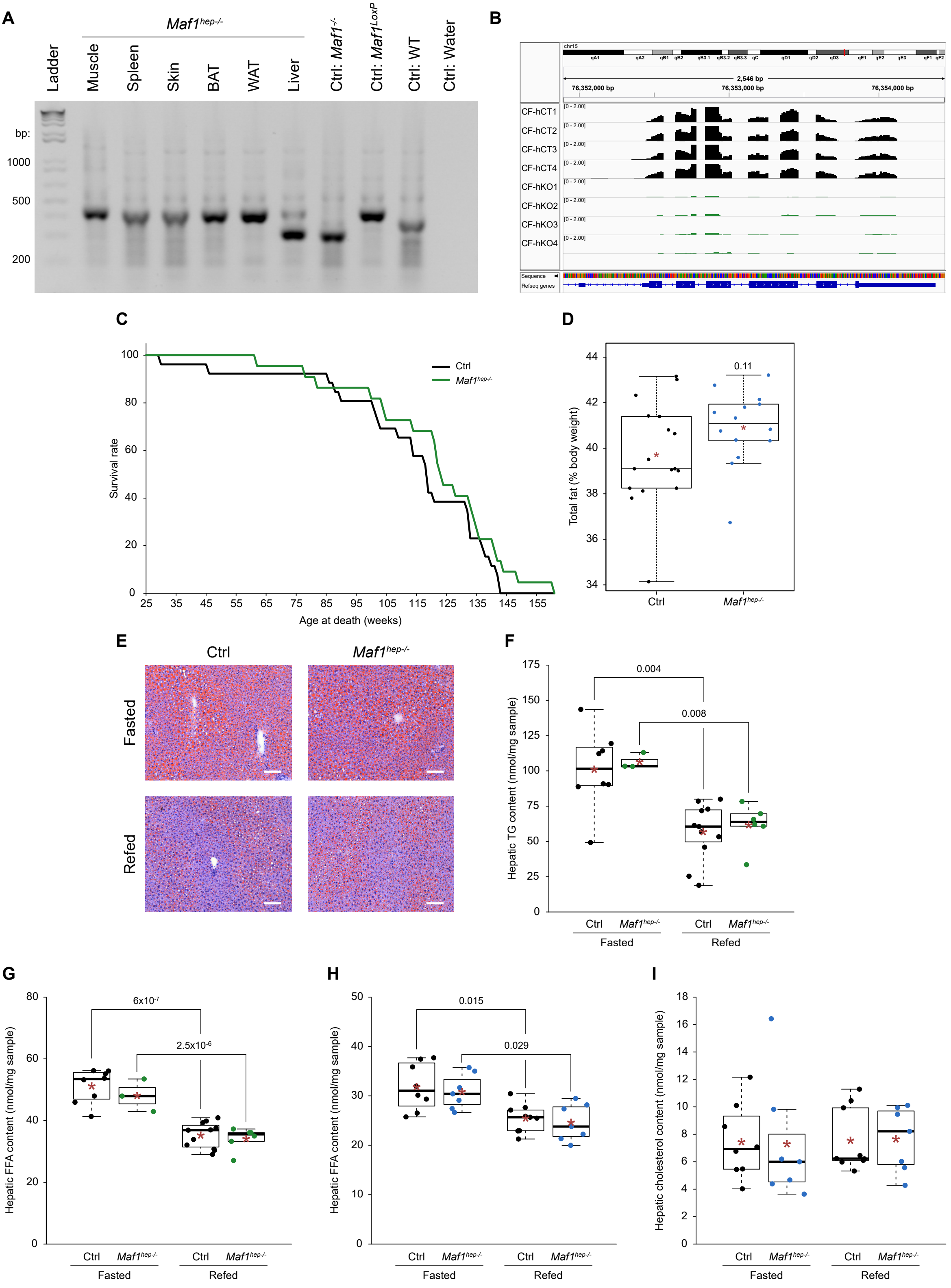

A
Maf1hep-/-
Ladder
Muscle
Spleen
Skin
BAT
WAT
Liver
Ctrl: Maf1-/-
Ctrl: Maf1LoxP
Ctrl: WT
Ctrl: Water
bp:
1000
500
200
B
chr15
2,546 bp
76,352,000 bp
76,353,000 bp
76,354,000 bp
CF-hCT1
CF-hCT2
CF-hCT3
CF-hCT4
CF-hKO1
CF-hKO2
CF-hKO3
CF-hKO4
[0 - 2.00]
Sequence
Refseq genes
C
Ctrl
Maf1hep-/-
Survival rate
Age at death (weeks)
D
0.11
Total fat (% body weight)
Ctrl
Maf1hep-/-
E
Ctrl
Maf1hep-/-
Fasted
Refed
F
0.004
0.008
Hepatic TG content (nmol/mg sample)
Ctrl
Maf1hep-/-
Ctrl
Maf1hep-/-
Fasted
Refed
G
6x10-7
2.5x10-6
Hepatic FFA content (nmol/mg sample)
Ctrl
Maf1hep-/-
Ctrl
Maf1hep-/-
Fasted
Refed
H
0.015
0.029
Hepatic FFA content (nmol/mg sample)
Ctrl
Maf1hep-/-
Ctrl
Maf1hep-/-
Fasted
Refed
I
Hepatic cholesterol content (nmol/mg sample)
Ctrl
Maf1hep-/-
Ctrl
Maf1hep-/-
Fasted
Refed

Figure S1

Figure S1. (A) Gel image of a genotyping PCR done on several *Maf1*$^{hep-/-}$ organs and specific Ctrl samples. PCR fragments sizes are 480bp for Maf1LoxP sites inclusion, 376bp for recombinated floxed *Maf1* knock out gene and 450bp for WT mice. As around 20% of liver cells are not hepatocytes, a Maf1LoxP fragment is expected in the liver sample. BAT: brown adipose tissue; WAT: white adipose tissue. **(B)** IGV genome browser view of the region encompassing the *Maf1* gene showing the number of RNAseq reads in Ctrl (black) and *Maf1*$^{hep-/-}$ (green) mice. **(C)** Kaplan-Meier survival curves of male Ctrl and *Maf1*$^{hep-/-}$ mice fed a Chow diet. Ctrl n=26 and *Maf1*$^{hep-/-}$ n=22. **(D)** Fat weight relative to total weight in 24 week-old Ctrl and *Maf1*$^{hep-/-}$ mice fed a HF diet. Ctrl n=17 and *Maf1*$^{hep-/-}$ n=14. **(E)** Representative neutral lipid Oil Red O staining of liver slices from 24 week-old fasted or refed Ctrl and *Maf1*$^{hep-/-}$ mice under a Chow diet. White bars: 100 µm. **(F)** Hepatic TG content (nmole/mg of tissue sample) in fasted or refed Ctrl and *Maf1*$^{hep-/-}$ mice under a Chow diet. Fasted Ctrl n=8 and *Maf1*$^{hep-/-}$ n=3; Refed Ctrl n=11 and *Maf1*$^{hep-/-}$ n=6. **(G)** Liver FFA content (nmol/mg of tissue sample) in the same samples as in **(E)**. **(H)** FFA content in the liver of fasted or refed Ctrl and *Maf1*$^{hep-/-}$ mice under a HF diet. Fasted Ctrl n=8 and *Maf1*$^{hep-/-}$ n=7; Refed Ctrl n=9 and *Maf1*$^{hep-/-}$ n=7. **(I)** Liver cholesterol content in the same samples as in **(H).** In **(D)** and **(F)** to **(I)**, the box encompasses upper and lower quartile, the whiskers extend to 1.5 times the interquartile range of the box. Red star, mean; horizontal bar, median.

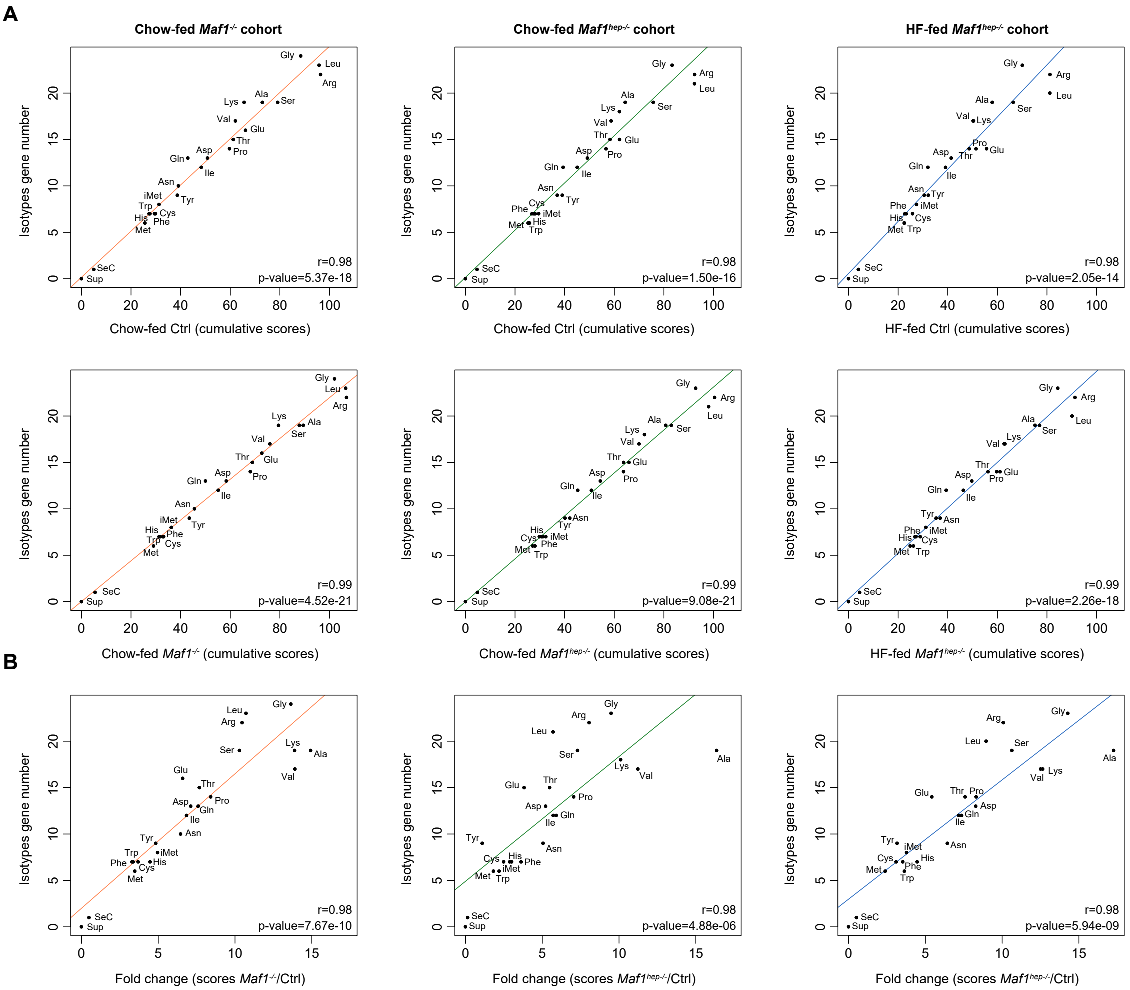

Figure S2. (A) Spearman rank correlations of Pol III occupancy cumulated scores for each tRNA isotype between Ctrl and *Maf1* deficient mice as indicated. The regression line is colored. **(B)** Spearman rank correlations between score fold change (for each cohort, Ctrl versus *Maf1* deficient mice) for each isotype (X axis) and number of corresponding isotype genes (Y axis), for Ctrl and *Maf1* deficient sample of each of the three cohorts, as indicated. The correlation line is colored.

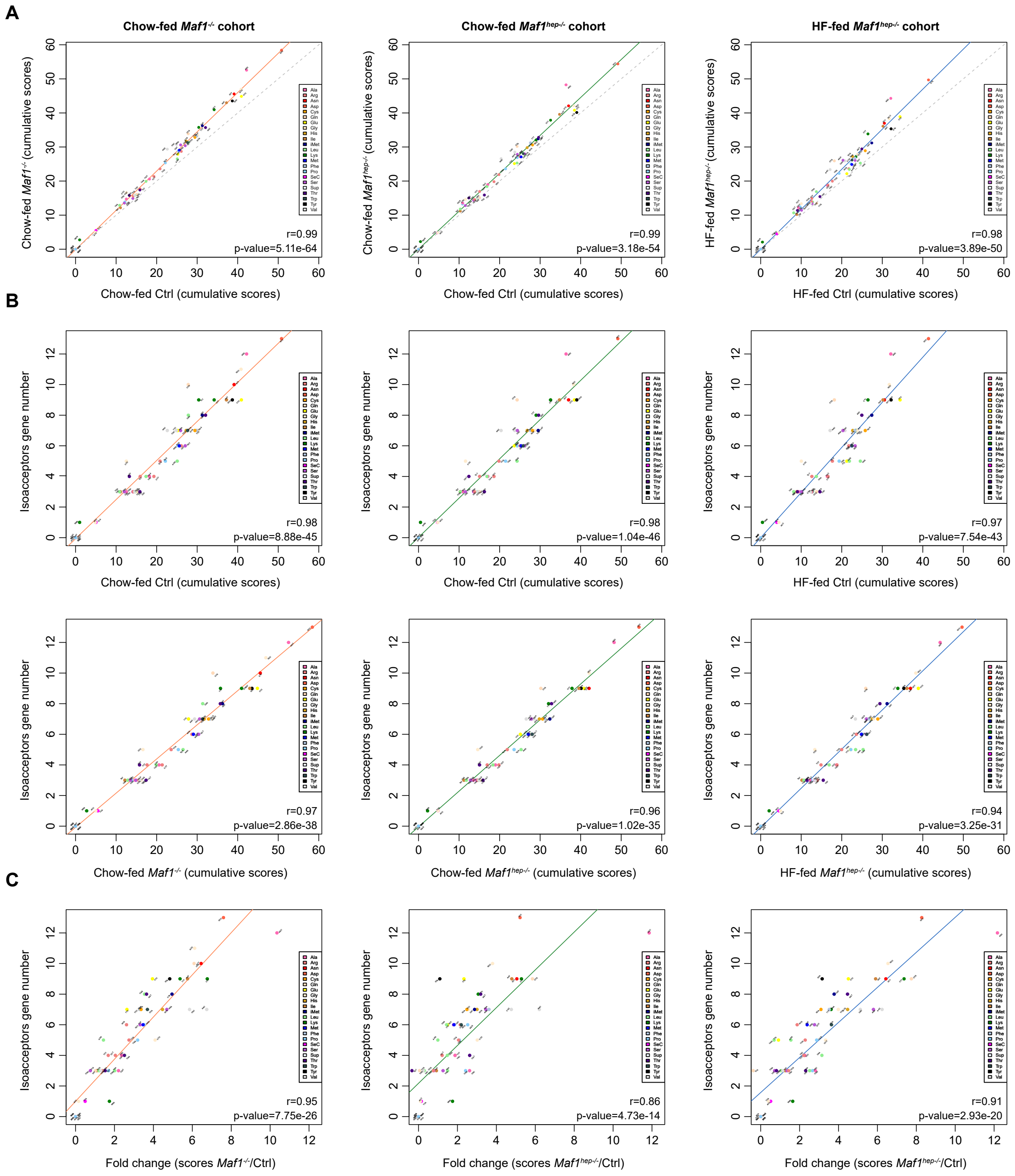
A
Chow-fed Maf1-/- cohort
Chow-fed Maf1hep-/- cohort
HF-fed Maf1hep-/- cohort
Chow-fed Maf1-/- (cumulative scores)
Chow-fed Maf1hep-/- (cumulative scores)
HF-fed Maf1hep-/- (cumulative scores)
Chow-fed Ctrl (cumulative scores)
HF-fed Ctrl (cumulative scores)
r=0.99
p-value=5.11e-64
r=0.99
p-value=3.18e-54
r=0.98
p-value=3.89e-50
B
Isoacceptors gene number
r=0.98
p-value=8.88e-45
r=0.98
p-value=1.04e-46
r=0.97
p-value=7.54e-43
r=0.97
p-value=2.86e-38
r=0.96
p-value=1.02e-35
r=0.94
p-value=3.25e-31
C
Fold change (scores Maf1-/-/Ctrl)
Fold change (scores Maf1hep-/-/Ctrl)
r=0.95
p-value=7.75e-26
r=0.86
p-value=4.73e-14
r=0.91
p-value=2.93e-20
Ala
Arg
Asn
Asp
Cys
Gln
Glu
Gly
His
Ile
iMet
Leu
Lys
Met
Phe
Pro
SeC
Ser
Sup
Thr
Trp
Tyr
Val

Figure S3

Figure S3. (A) Spearman rank correlations of Pol III occupancy cumulated scores for each tRNA isoacceptor between Ctrl and *Maf1* deficient mice as indicated. The regression line is colored, the x=y line is dotted. **(B)** Spearman rank correlations between cumulated scores for each isoacceptor (X axis) and number of corresponding isoacceptor genes (Y axis). The correlation line is colored. **(C)** Spearman rank correlations between score fold change (for each cohort, Ctrl versus *Maf1* deficient mice) for each isoacceptor (X axis) and number of corresponding isoacceptor genes (Y axis), for Ctrl and *Maf1* deficient sample of each of the three cohorts, as indicated. The correlation line is colored.

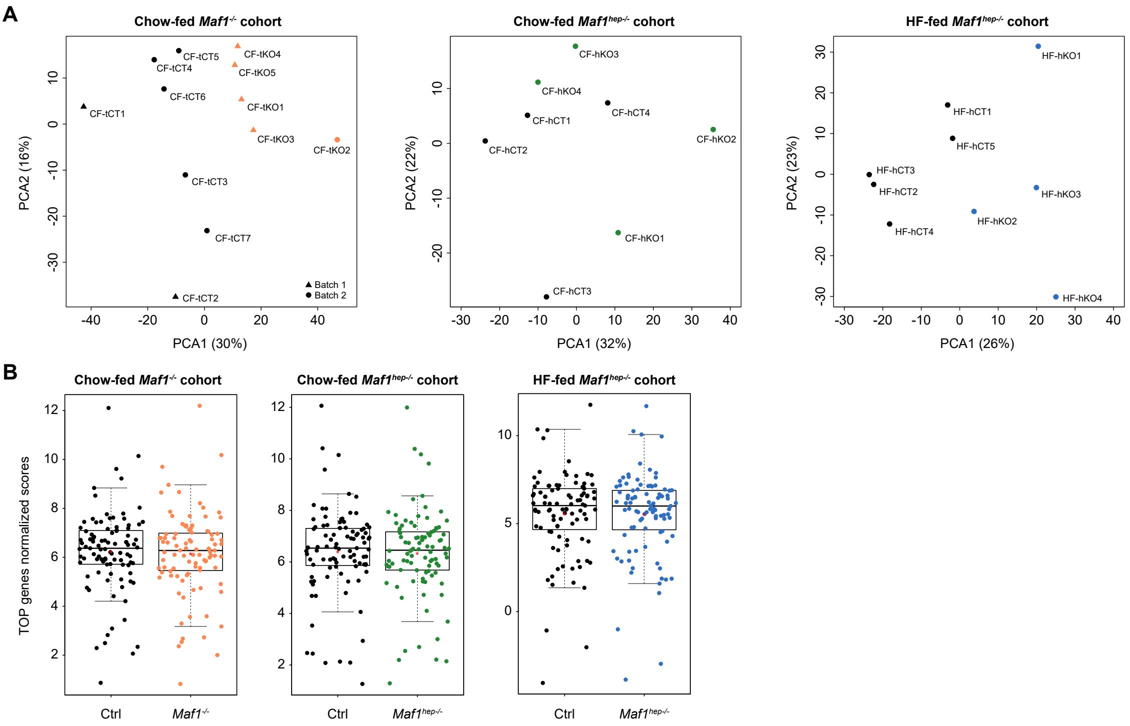

Figure S4. **(A)** Principal component analysis (PCA) of the samples used for RNAseq. Black symbols: Ctrl samples. Colored symbols: knockout samples from Chow-fed *Maf1*$^{-/-}$ (orange), Chow-fed *Maf1*$^{hep-/-}$ (green), and HF-fed *Maf1*$^{hep-/-}$ (blue) mice. For the Chow-fed *Maf1*$^{-/-}$ cohort, dots and triangles indicate 2 different batches of data acquired at different times. **(B)** Box plots indicating the scores of TOP mRNAs for indicated mice. The box encompasses upper and lower quartile, the whiskers extend to 1.5 times the interquartile range of the box. Red star, mean; horizontal bar, median.

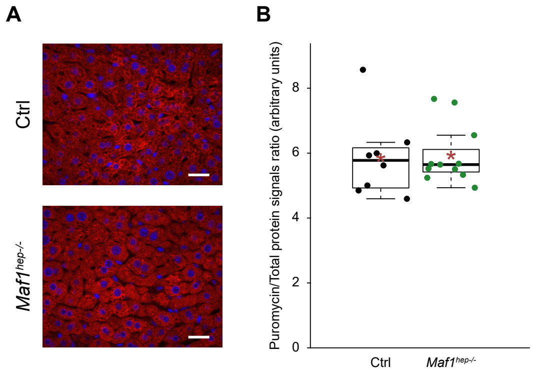

Figure S5. (A) Representative puromycin and DAPI staining of liver slices from 13-14 week-old Chow-fed *Maf1*$^{hep-/-}$ mice. White bars represent 20 µm. **(B)** WES™ quantification of puromycin protein incorporation in the livers of mice from the same Chow-fed *Maf1*$^{hep-/-}$ cohort. Ctrl n=8 and *Maf1*$^{hep-/-}$ n=11. The boxes encompass upper and lower quartile, the whiskers extend to 1.5 times the interquartile range of the box. Red star, mean; horizontal bar, median.
